# Preferential Inhibition of Wnt/β-Catenin Signaling by Novel Benzimidazole Compounds in Triple-Negative Breast Cancer

**DOI:** 10.3390/ijms19051524

**Published:** 2018-05-20

**Authors:** Abhishek Gangrade, Vibha Pathak, Corinne E. Augelli-Szafran, Han-Xun Wei, Patsy Oliver, Mark Suto, Donald J. Buchsbaum

**Affiliations:** 1 Department of Radiation Oncology, University of Alabama at Birmingham, Birmingham, AL 35294, USA; abhigan8@gmail.com; 2 Department of Chemistry, Drug Discovery Division, Southern Research, Birmingham, AL 35205, USA; vpathak@southernresearch.org (V.P.); caugelli-szafran@southernresearch.org (C.E.A.-S.); hweihml@hotmail.com (H.-X.W.); msuto@southernresearch.org (M.S.); 3 Division of Pulmonary, Allergy and Critical Care, University of Alabama at Birmingham, Birmingham, AL 35233, USA; poliver@uab.edu

**Keywords:** receptor-targeted therapy, LRP6, triple-negative breast cancer, Wnt/β-catenin signaling, benzimidazoles

## Abstract

Wnt/β-catenin signaling is upregulated in triple-negative breast cancer (TNBC) compared to other breast cancer subtypes and normal tissues. Current Wnt/β-catenin inhibitors, such as niclosamide, target the pathway nonspecifically and exhibit poor pharmacokinetics/pharmacodynamics in vivo. Niclosamide targets other pathways, including mTOR, STAT3 and Notch. Novel benzimidazoles have been developed to inhibit Wnt/β-catenin signaling with greater specificity. The compounds SRI33576 and SRI35889 were discovered to produce more cytotoxicity in TNBC cell lines than in noncancerous cells. The agents also downregulated Wnt/β-catenin signaling mediators LRP6, cyclin D1, survivin and nuclear active β-catenin. In addition, SRI33576 did not affect mTOR, STAT3 and Notch signaling in TNBC and noncancerous cells. SRI35889 inhibited mTOR signaling less in noncancerous than in cancerous cells, while not affecting STAT3 and Notch pathways. Compounds SRI32529, SRI35357 and SRI35361 were not selectively cytotoxic against TNBC cell lines compared to MCF10A cells. While SRI32529 inhibited Wnt/β-catenin signaling, the compound also mitigated mTOR, STAT3 and Notch signaling. SRI33576 and SRI35889 were identified as cytotoxic and selective inhibitors of Wnt/β-catenin signaling with therapeutic potential to treat TNBC in vivo.

## 1. Introduction

Of all cancers, breast cancer affects women the most with an incidence of about 12%. High morbidity of this disease is associated with triple-negative breast cancer (TNBC), which accounts for 10–20% of all breast cancers [1,2,3] The moniker of the disease derives from lack of progesterone, estrogen and HER2 receptors, commonly targeted by chemotherapy, hormone therapy and HER2 antibody. As a result, these cancers exhibit drug resistance [4]. Known as the most aggressive breast cancer subtype, TNBC requires new targeted therapies to overcome resistance and lessen probability of relapse.

Underlying signaling characteristics of TNBC may be exploited for developing novel therapies. Wnt/β-catenin signaling drives tumorigenesis in a variety of cancers and contributes to progression of TNBC compared to that of other breast cancer subtypes [5,6,7,8]. Wnt/β-catenin signaling is activated upon binding of secreted cysteine-rich Wnt glycoproteins to LRP5/6 and Frizzled co-receptors, leading to inactivation of β-catenin-phosphorylating protein GSK3β. Lack of phosphorylation enables β-catenin to avoid ubiquitination and degradation, thus allowing the protein to translocate to the nucleus, bind to TCF/LEF transcription factors and promote transcription of target genes, such as cyclin D1 and survivin, that drive tumorigenesis and cell proliferation [9,10,11,12].

Unlike colorectal cancers, of which 80–90% contain APC mutations, TNBC generally lacks mutations in Wnt/β-catenin pathway related proteins. Instead, pathway dysregulation is mediated by activation of β-catenin-mediated transcription [7,13] and high expression of Wnt/β-catenin pathway mediators such as Frizzled [14,15] and LRP6 [16]. Both autocrine and paracrine Wnt/β-catenin signaling are implicated in TNBC progression [17,18,19]. LRP6 surface receptor is overexpressed in triple-negative, ER-negative and HER2-negative breast cancers. At the genetic level, LRP6 expression is more than 6-fold higher in 6 of 7 TNBC cell lines compared to noncancerous MCF10A cells [16]. At the protein level, LRP6 was overexpressed in 4 of 7 TNBC cell lines compared to MCF10A cells. Little overlap was observed between tumor samples displaying higher LRP6 transcript and those with higher HER2 transcript, signifying that Wnt/β-catenin signaling is possibly an independent diagnostic indicator [16]. Inhibition of LRP6 expression and/or activity with Mesd, an LRP6 antagonist, in MDA-MB-231 cells decreased cell viability, proliferation and colony formation [16]. In addition, inhibition of LRP6 activity led to decrease in migration and invasion of TNBC cells. Treatment with Mesd also decreased expression of S100A4, a Wnt/β-catenin signaling target and a contributor to cancer metastasis [20]. As a result, targeting LRP6 represents an appealing therapeutic strategy for TNBC. Blood of mice dosed with 5, 10, 20 mg/kg Mesd had the highest concentration after 2 h. Tissue distribution of Mesd was rapid and widespread as the bioavailability averaged about 60% [16].

Novel small molecule inhibitors targeting Wnt/β-catenin signaling in cancers have been recently developed [21]. Appealing targets have included CREB-binding protein (CBP), a coactivator involved in β-catenin-mediated transcription by binding either β-catenin or TCF [22] and porcupine, a Wnt-modifying enzyme [23]. Currently, agents such as PRI-724, a CBP suppressor and WNT974, a porcupine inhibitor, are undergoing investigation for use as sole agents in phase I clinical trials in TNBC patients [24]. Small molecule inhibitor CWP232228 suppressed β-catenin-mediated transcription in TNBC by mitigating β-catenin/TCF interaction, inhibiting stem cell proliferation and lowering tumor bulk [25]. The agent displayed minimal toxicity and no obvious clinical signs in animals. CWP232228, administered IV at a dose of 200 mg/kg yielded a blood concentration of 0.8 µg/mL for 7 h [25]. Inhibitors such as XAV939 [26] and IWP-2 [27] have been shown to target Wnt/β-catenin activity via in vitro studies, although they exhibit suboptimal pharmacokinetic and pharmacodynamic (PK/PD) characteristics. As a result, Wnt/β-catenin pathway inhibitors with improved PK/PD activities are required for more promising therapies in vivo.

Maintenance of cancer stem cells (CSCs), a subpopulation of cells involved in self-renewal and regeneration of tumors, has been associated with Wnt/β-catenin signaling. Breast CSCs, identified by CD44+/CD24− markers, have the capacity to generate tumors [28,29]. High β-catenin expression correlates with the CD44+/CD24− phenotype [30]. In addition, suppression of WNT1 changes the phenotype and lessens tumor formation and cell migration [31]. Attenuation of Wnt/β-catenin signaling due to inhibition of protein kinase D1 (PKD1) decreased CSC characteristics in breast cancer cells [32].

High-throughput screening of more than 4000 clinically approved compounds revealed the anthelmintic agent niclosamide as a potent Wnt/β-catenin signaling inhibitor with anticancer activity [33]. Currently undergoing clinical trials in colorectal and prostate cancers, niclosamide inhibits proliferation of various cancers at concentrations that also inhibit Wnt/β-catenin signaling activities [34,35]. Niclosamide inhibited Wnt/β-catenin signaling in TNBC by inducing LRP6 degradation, enabling Frizzled1 internalization and mitigating interaction between β-catenin and TCF [34,35,36,37]. Anthelmintic activities of niclosamide have been shown to influence ATP homeostasis and uncoupling of oxidative phosphorylation [38,39]. Potentially, niclosamide may inhibit Wnt/β-catenin signaling and elicit anthelmintic effects via different mechanisms [40]. In addition, the drug has also been found to impact mTOR, STAT3 and Notch signaling in various cancers [41,42,43,44,45,46]. Our laboratory has previously shown niclosamide affects both Wnt/β-catenin and STAT3 signaling pathways in bulk and stem TNBC cells [47]. Niclosamide has shown some efficacy after systemic administration against various cancers in animal studies but the agent is not well absorbed from the gastrointestinal tract following oral administration as used for treatment of tapeworm, attributed to its weak metabolic stability (t_1/2_ = 29 min in rat liver microsomes) and solubility (1.6 µM, pH 7.4). Ideally, the pharmacokinetic t_1/2_ should be at least 60 min, requiring twice daily dosing.

Benzimidazole compounds (agents featuring fused benzene and imidazole rings) have been evaluated for anticancer effects [40,48,49,50]. Due to the poor bioavailability of niclosamide, several benzimidazole compounds were developed by modifying the structure of niclosamide to improve in vivo potential. The amide group of niclosamide was constrained to generate the five-membered ring scaffold for benzimidazole analogs used in this study. A class of compounds, known as 2,5-disubstituted phenyl benzimidazoles, produced cytotoxic and Wnt/β-catenin-inhibiting effects. These compounds are characterized by substituents located in the 2 and 5 positions of the phenyl ring in benzimidazoles. The NO_2_ group in niclosamide, known to elicit toxic effects, was removed from most of the inhibitors. Since niclosamide was postulated to affect Wnt/β-catenin signaling separately from ATP homeostasis, Mook et al. (Duke University) developed novel compounds to improve Wnt/β-catenin selectivity. The structure of a selective compound evaluated in this study, SRI33576, was first identified by Mook et al. to have specificity for Wnt/β-catenin signaling over ATP homeostasis in colorectal cancer cells [40]. SRI32529 was first developed in a screen of thirty 6-nitrobenzimidazoles for phosphodiesterase inhibitors. SRI32529 was not selected as one of the effective inhibitors [51]. In addition, SRI32529 was found to have less potency in inhibiting Wnt/β-catenin signaling than SRI33576 [40]. Neither compound has been evaluated for antiproliferative effects in cancer cells. Also, the effects of SRI33576 have not been distinguished from those of niclosamide on non-Wnt/β-catenin signaling pathways, such as mTOR, STAT3 and Notch. Benzimidazole compounds developed at Southern Research (SR) were screened for effects on cell viability and apoptosis in TNBC and selectivity for Wnt/β-catenin signaling. SRI33576 and SRI35889 were discovered to have more cytotoxic efficacy and specificity against Wnt/β-catenin signaling in TNBC than in noncancerous cells.

In the current study, modification of niclosamide has led to the identification of novel benzimidazole compounds that inhibit Wnt/β-catenin signaling. SRI33576 and SRI35889 are effective and selective Wnt/β-catenin inhibitors which do not affect mTOR, STAT3 and Notch signaling. We propose these compounds are potentially effective therapeutic agents for cancers dependent on inhibition of Wnt/β-catenin signaling.

## 2. Results

### 2.1. SRI33576 and SRI35889 are More Cytotoxic to TNBC Than to Noncancerous MCF10A Cells

**Figure 1:**
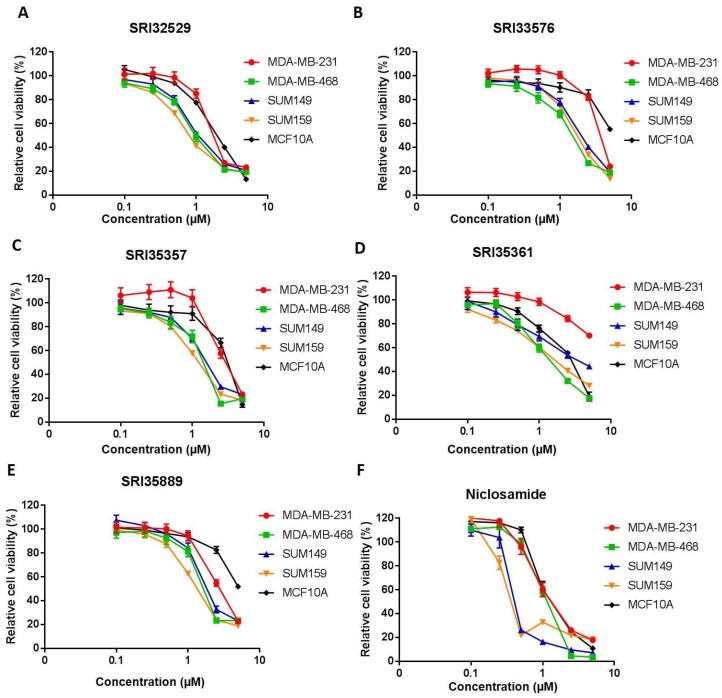
Effects of benzimidazole compounds on viability of triple negative breast cancer (TNBC) and noncancerous MCF10A cells. Cells were treated with 0–5 µM SRI32529 (**A**), SRI33576 (**B**), SRI35357 (**C**), SRI35361 (**D**), SRI35889 (**E**) and niclosamide (**F**) for 72 h. Assays were performed three times.

Five of the 2,5-disubstituted phenyl benzimidazoles were tested for cytotoxicity and inhibition of Wnt/β-catenin signaling (Table 1). First, it was important to compare efficacies of the SR compounds in inhibiting proliferation of TNBC and noncancerous cells. Both SRI33576 and SRI35889 exhibit more cytotoxicity in TNBC cell lines than in noncancerous MCF10A cells. IC_50_ values for SUM149, SUM159, MDA-MB-231 and MDA-MB-468 TNBC cell lines treated with SRI33576 ranged from 1.9 to 3.2 µM, compared to 5.0 µM for MCF10A cells. IC_50_ values for TNBC cell lines treated with SRI35889 ranged from 1.1 to 2.4 µM, compared to 5.4 µM for treated MCF10A cells (Figure 1). IC_50_ values for TNBC cell lines treated with niclosamide, an inhibitor of multiple signaling pathways, ranged from 0.3 to 1.0 µM, while the IC_50_ value for MCF10A cells was 1.0 µM. Thus, compared to SR agents, niclosamide exhibits less selectivity in eliciting cytotoxicity in TNBC cells versus noncancerous cells.

### 2.2. SRI33576 and SRI35889 Induce Apoptosis in TNBC

**Figure 2:**
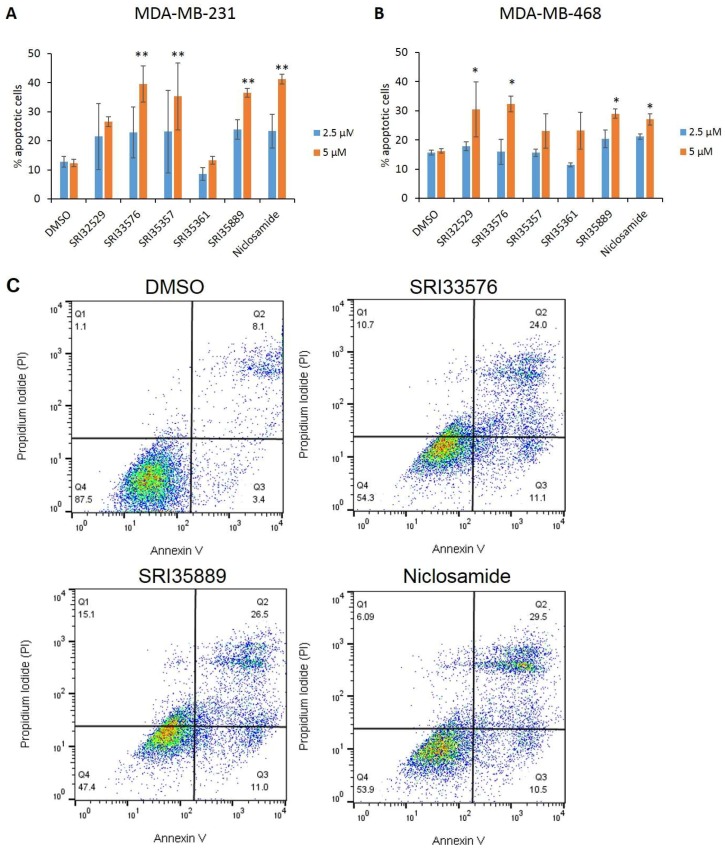
Apoptosis of (**A**) MDA-MB-231 and (**B**) MDA-MB-468 TNBC cells following treatment with Southern Research (SR) compounds and niclosamide for 48 h. Apoptosis was measured by staining cells with Annexin V and PI prior to analysis by flow cytometry. Apoptosis of cells treated with compounds was compared to DMSO-treated cells. (**C**) Representative scatterplots of MDA-MB-231 cells treated with DMSO, 5 µM SRI33576, SRI35889 and niclosamide for 48 h. Assays were performed three times. * *p* < 0.05, ** *p* < 0.01.

Apoptosis of TNBC cell lines was evaluated by Annexin V/PI assay following treatment with the agents. Compounds SRI33576 and SRI35889 induced significantly more apoptosis in MDA-MB-231 and MDA-MB-468 cells treated with 5 µM for 48 h. SRI35361 was the only compound which did not induce significantly more apoptosis than DMSO in either cell line (Figure 2A,B). In addition, a higher percentage of MDA-MB-231 cells treated with 5 µM SRI33576 (24% of total) and SRI35889 (26.5%) resided in the late apoptotic phase as compared to vehicle control-treated cells (8.1%). This was comparable to cells treated with niclosamide (29.5%) as shown in Figure 2C.

### 2.3. SRI33576 and SRI35889 Inhibit Wnt/β-Catenin Signaling in TNBC Cell Lines But Not Noncancerous Cells

**Figure 3:**
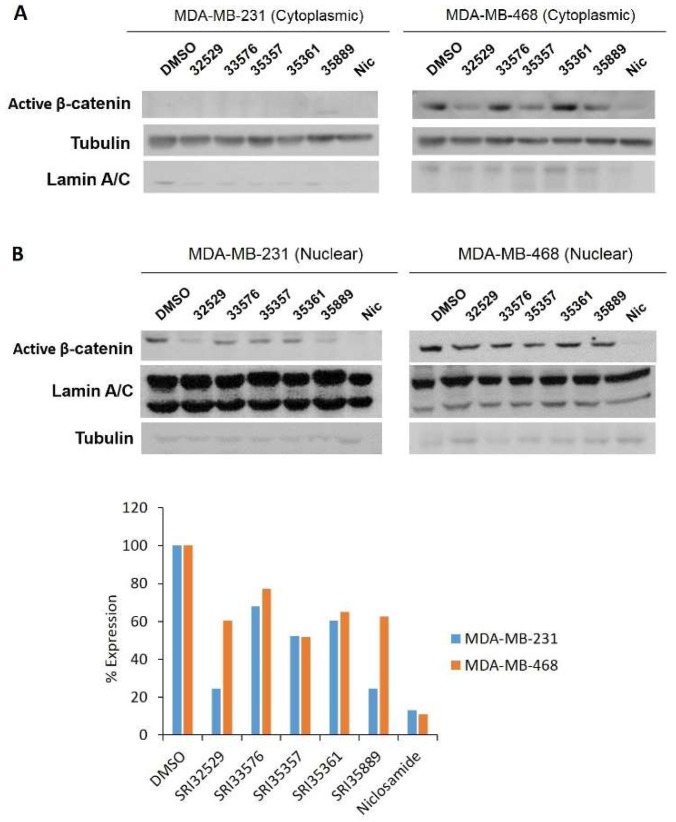
Effects of SR compounds and niclosamide on active β-catenin expression in TNBC cells. (**A**) Cytoplasmic and (**B**) nuclear active β-catenin expression was evaluated in the cells following 18 h treatment with 5 µM concentrations of the compounds and cytoplasmic-nuclear fractionation. Intensity of nuclear active β-catenin bands in (**B**) were quantified by densitometry with ImageJ software and normalized to corresponding band of lamin A/C. Western blotting was performed on lysates (40 ug loaded). Active β-catenin was not detected in cytoplasm of MDA-MB-231 cells. Tubulin and lamin A/C were detected to ensure proper separation of cytoplasmic and nuclear fractions. Western blotting was performed two times.

**Figure 4:**
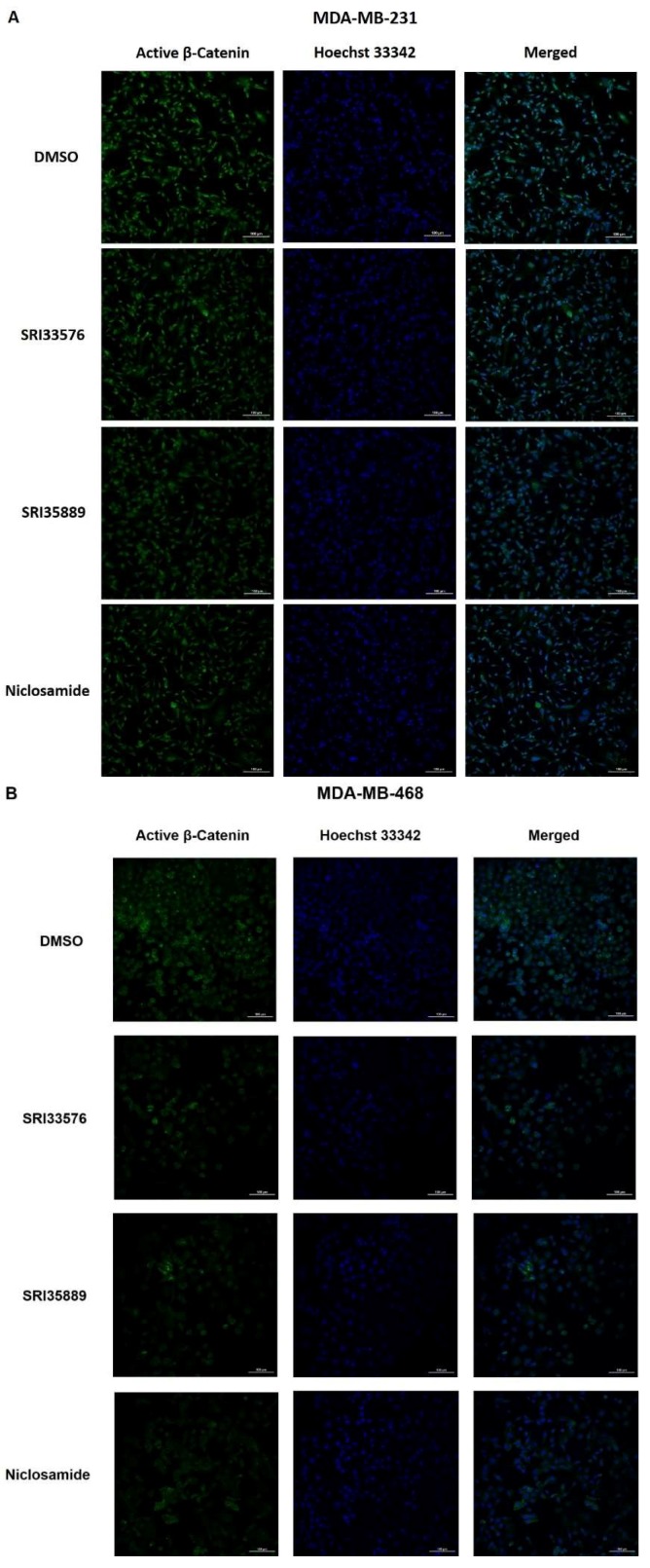

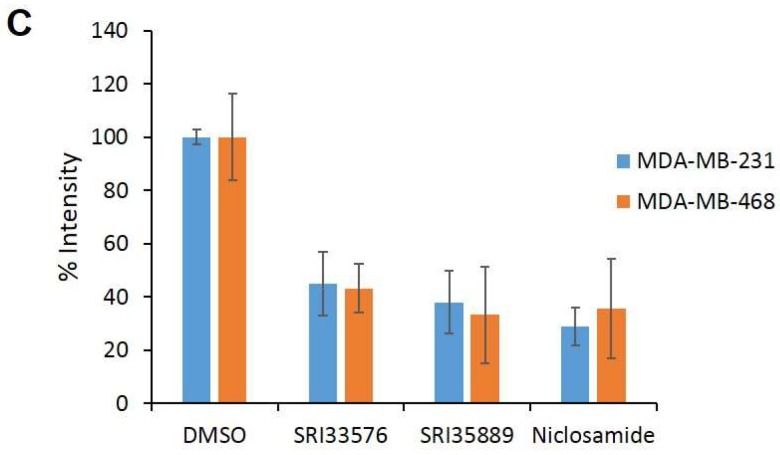
Immunocytochemistry (ICC) of active β-catenin following treatment with SR compounds. IF of active β-catenin in (**A**) MDA-MB-231 and (**B**) MDA-MB-468 cells treated with 0.05% DMSO, 5 µM SRI33576, SRI35889 and niclosamide. Cells were counterstained with Hoechst 33342 (blue). (**C**) Fluorescence intensities were quantified by ImageJ and means were compared to DMSO-treated cells (*n* = 3). Scale bar = 100 µm.

We sought to determine specificity of the two compounds for inhibition of Wnt/β-catenin signaling in TNBC over noncancerous cells. As niclosamide is an inhibitor of several pathways, we compared the effects of this agent with the SR benzimidazoles. Modulation of Wnt/β-catenin pathway-related proteins by the compounds was detected by Western blotting. Nuclear β-catenin promotes transcription of pro-tumorigenesis genes and cytoplasmic β-catenin is associated with poor prognosis in breast cancer patients [52]. To detect cytoplasmic/nuclear active (non-phosphorylated) β-catenin, we used a monoclonal antibody which recognizes non-phosphorylated sites Ser-37 and Thr-41. The antibody has been shown to visualize production of active β-catenin via the canonical Wnt/β-catenin pathway during murine embryogenesis [53]. Interestingly, active β-catenin was observed in the cytoplasm of MDA-MB-468 cells but not MDA-MB-231 cells. SRI35889 decreased cytoplasmic β-catenin (Figure 3A). SRI33576 and SRI35889 decreased nuclear active β-catenin expression following 18 h treatment (Figure 3B). Immunocytochemistry also revealed a decrease in nuclear active β-catenin expression with SRI33576 and SRI35889 treatment in MDA-MB-231 and MDA-MB-468 cells (Figure 4).

**Figure 5:**
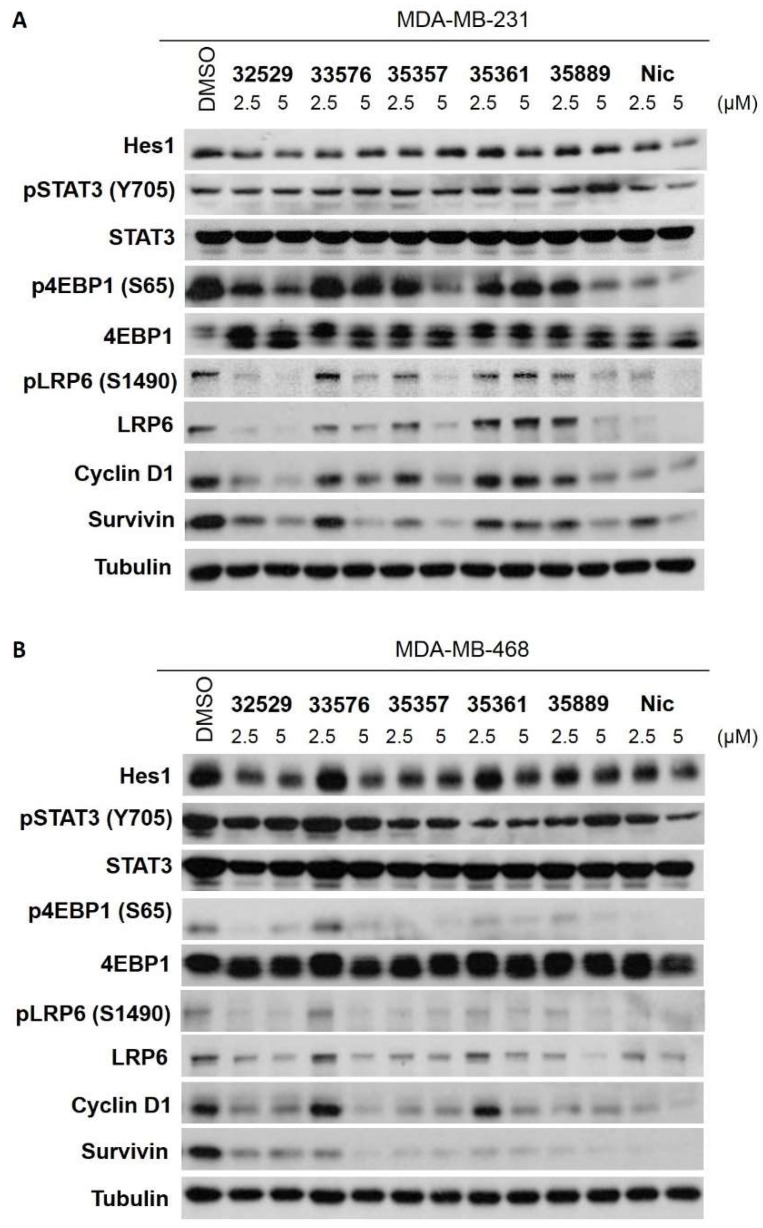

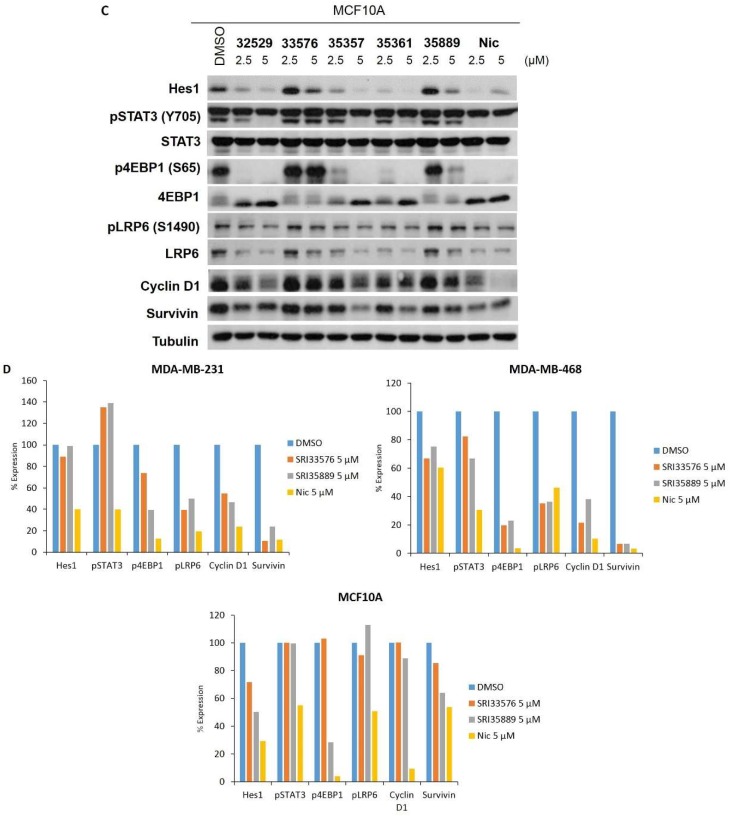
Effect of SR compounds on Wnt/β-catenin signaling in (**A**) MDA-MB-231, (**B**) MDA-MB-468 and (**C**) MCF10A cells treated with 2.5 or 5 µM of compounds for 18 h. Hes1, pSTAT3 (Y705), STAT3, p4EBP1 (p65), 4EBP1, pLRP6, cyclin D1, survivin and tubulin in whole cell lysates were detected by immunoblotting. (**D**) Densitometry readings for select proteins Hes1, pSTAT3, p4EBP1, pLRP6, LRP6, cyclin D1 and survivin according to Western blots. Results are averages of two replicate experiments and normalized to tubulin. 0.05% DMSO was used as vehicle control. Western blotting was performed two times.

Because the receptor LRP6 is an initiator of Wnt/β-catenin signaling, we sought to assess whether the compounds inhibited expression of the phosphorylated (active) form. SRI33576 decreased phosphorylated LRP6 at 5 µM in both TNBC cell lines but not MCF10A cells. SRI35889 decreased phosphorylated LRP6 at 2.5 and 5 µM in both TNBC cell lines but not MCF10A cells. Niclosamide inhibited decreased phosphorylated LRP6 in MCF10A cells (Figure 5).

### 2.4. SRI33576 and SRI35889 Have Less Effect on mTOR, STAT3 and Notch Signaling Than Niclosamide

Niclosamide affects mTOR, STAT3 and Notch pathways, potentially conferring increased toxicity in normal tissues. Specificity of benzimidazole compounds was evaluated by assessing their effects on non-Wnt/β-catenin signaling pathways mTOR, STAT3 and Notch. Cell lines were treated with the compounds and assayed by Western blotting for changes in protein levels associated with these pathways. Niclosamide downregulated STAT3 phosphorylation in TNBC and MCF10A cells, whereas SRI33576 and SRI35889 produced noticeably less of an effect (Figure 5). SRI33576 inhibited phosphorylation of 4EBP1 at 5 µM in both TNBC cell lines but it did not yield such a change in MCF10A cells. SRI35889 inhibited 4EBP1 phosphorylation at 2.5 and 5 µM in both TNBC cell lines but it produced less of a change in MCF10A cells at 2.5 µM. Neither of the compounds downregulated phosphorylation of 4EBP1 as much as niclosamide (Figure 5). Expression of the pSTAT3β and total STAT3β isoform (lower band), associated with tumor-suppressor function [54], was markedly decreased by niclosamide but not by SRI33576 or SRI35889 in MDA-MB-468 and MCF10A cells. SRI33576 and SRI35889 decreased expression of Wnt/β-catenin pathway proteins cyclin D1 and survivin in TNBC cells at 2.5 and 5 µM concentrations. At 2.5 µM concentration, SRI35889 did not affect cyclin D1 or survivin expression as much as niclosamide in MCF10A cells (Figure 5).

## 3. Discussion

Despite the fact Wnt/β-catenin signaling is not driven by mutations encoding pathway-related proteins, Wnt/β-catenin signaling represents an appealing target for inhibitors due to higher activity in TNBC than in normal tissues. Niclosamide has been established as an effective inhibitor of Wnt/β-catenin signaling in several cancers, including colon, breast and myeloma [36,55,56,57,58]. Studies indicated niclosamide exhibits no carcinogenic effects in animals [38]. However, several studies have shown the agent elicits genotoxic effects [59,60,61]. SRI33576 was previously found to inhibit Wnt/β-catenin signaling in colorectal cancer cells by decreasing cytosolic axin2 and β-catenin and overall c-myc, survivin and cyclin D1 levels [40]. However, it was not known whether nuclear β-catenin, the major driver of canonical Wnt/β-catenin signaling, was abrogated by the agent in colorectal cancer cells. To our knowledge, this is the first study evaluating the effects of 2,5-disubstituted phenyl benzimidazoles on TNBC cell lines.

Benzimidazoles SRI33576 and SRI35889 were identified with improved specificity for Wnt/β-catenin signaling in TNBC cell lines than in noncancerous cells. Compared to niclosamide, both agents preferentially inhibit Wnt/β-catenin signaling, indicated by lower expression of Wnt/β-catenin pathway proteins survivin and cyclin D1. A member of the inhibitor of apoptosis (IAP) family, survivin controls cell division, mitigates apoptosis and suppresses active caspases [62,63,64,65,66]. Overexpression of cyclin D1, a driver of G1/S transition during cell cycle progression, has been observed in more than half of breast cancers [67]. Inhibition of Wnt/β-catenin signaling in TNBC is associated with promotion of apoptosis [5]. Both agents increased apoptotic levels in TNBC cells, potentially due to their effects on Wnt/β-catenin signaling. In addition, it appears that compounds SRI33576 and SRI35889 decrease active β-catenin expression via targeting of LRP6 in TNBC cells over noncancerous MCF10A cells. As TNBC is characterized by high LRP6 expression [16,20], targeting of this receptor by the compounds underscores their therapeutic relevancy. In addition, the inhibitors exhibited less pronounced effects on mTOR signaling in TNBC cell lines than in noncancerous cells. Neither SRI33576 nor SRI35889 affected Notch or STAT3 signaling activities in TNBC cell lines.

It is known autocrine Wnt/β-catenin signaling drives TNBC cell proliferation [17]. Both SRI33576 and SRI35889 inhibited autocrine Wnt/β-catenin signaling using established human cancer cell lines. However, paracrine signaling via activity of ligands such as Wnt3a may also play a role in TNBC growth [19] and it remains to be elucidated whether SRI33576 and SRI35889 can inhibit this pathway.

We previously evaluated other novel analogs of niclosamide that inhibited Wnt/β-catenin and mTOR signaling [68,69]. SRI35889 and to a lesser extent, SRI33576, were found to inhibit mTOR signaling in addition to Wnt/β-catenin activity, which may confer increased cytotoxic potency in TNBC. Recently, a study supported the use of dual inhibition of both PI3K/AKT/mTOR and Wnt/β-catenin pathways for synergistic effects in TNBC [70]. Interestingly, treatment of TNBC with the pan-PI3K inhibitor buparlisib increased Wnt/β-catenin activity via increased expression of Wnt/β-catenin pathway mediators such as FZDs, Wnt ligands, LRP4/6 and porcupine. Unfortunately, the inhibitor was found to elicit toxic effects, such as anxiety, pneumonititis and liver toxicity, in humans.

An appealing target of new therapies, CSCs participate in tumor recurrence, metastasis and resistance to chemotherapies in breast cancer. Evidence indicates β-catenin expression correlates with chemoresistance of TNBC as β-catenin knockdown sensitized TNBC cell lines to doxorubicin- or cisplatin-mediated cell death [13]. Wnt/β-catenin pathway inhibitors niclosamide, its analogs and salinomycin were found to effectively target breast and ovarian CSCs [47,69,71,72,73,74,75]. Because Wnt/β-catenin signaling mediates self-renewal and migration of CSCs, SRI33576 and SRI35889 may represent clinically useful therapeutic agents.

This study supports SRI33576 and SRI35889 for further investigation in animal studies. These compounds must be further evaluated for their PK/PD properties to elucidate their potential as cancer-targeting agents. In addition, both compounds may be combined with chemotherapy or immunotherapeutic agents to further enhance their therapeutic effects.

## 4. Materials and Methods

### 4.1. Compound Synthesis

**Figure 6:**
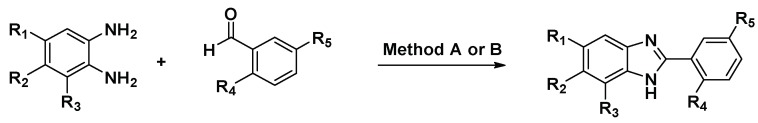
Synthesis of compounds SRI32529 and SRI33576. Method A. NaHSO_3_, DMSO, 210 °C, 1 h; Method B: Na_2_S_2_O_5_. DMF, 170 °C, 15 min.

Synthesis of compounds SRI32529 and SRI33576 have been previously described [40,51], Figure 6.

Method A utilized the condensation of diamines and aldehydes in presence of sodium sulfite and DMSO at 210 °C for 1 h. An improved synthetic method (Method B) was determined which utilized microwave conditions in the reaction of the substituted diamines and aryl rings in the presence of sodium metabisulfite and dry DMF. Method B was preferred versus Method A due to shorter reaction times (15 min) and higher yields.

#### 4.1.1. Method A

A mixture of an appropriate 1,2-phenylenediamine (1.0 eq), appropriate aldehyde (1.0 eq) and sodium bisulfite (1.0 eq) in 10 mL of DMSO was heated at 210 °C for 1 h. The reaction mixture was filtered, concentrated in vacuo and purified on a purification system using 10–50% hexanes and EtOAc.

#### 4.1.2. Method B

A mixture of an appropriate 1,2-phenylenediamine (1.0 eq), appropriate aldehyde (1.0 eq) and sodium metabisulfite (1.0 eq) in 8 mL of DMF was heated in a CEM microwave at 170 °C for 15 min. The reaction mixture was filtered, concentrated in vacuo and purified on a purification system using 10–50% hexanes and EtOAc.

#### 4.1.3. 2-(5,7-Dichloro-3*H*-benzo[*d*]imidazol-2-yl)-4-fluorophenol (35357)

This compound was prepared from 3,5-dichlorobenzene-1,2-diamine and 5-fluoro-2-hydroxybenzaldehyde using Method A. Yield 21%. TLC R_f_ = 0.40 (Hexanes–EtOAc, 2:1). ^1^H NMR (400 MHz, DMSO-*d*_6_) δ 13.40 (s, 1H), 7.95 (s, 1H), 7.71 (s, 1H), 7.48 (s, 2H), 7.30 (ddd, *J* = 9.2, 8.1, 3.1 Hz, 1H), 7.09 (dd, *J* = 9.1, 4.7 Hz, 1H). HRMS *m*/*z* calcd. for C_13_H_7_Cl_2_FN_2_O [M + H]^+^: 296.9992, found: 296.9993.

#### 4.1.4. 2-(5-Chloro-6-fluoro-1*H*-benzo[*d*]imidazol-2-yl)-4-fluorophenol (35361). 

This compound was prepared from 4-chloro-5-fluorobenzene-1,2-diamine and 5-fluoro-2-hydroxybenzaldehyde using Method A. Yield 64%. TLC R_f_ = 0.45 (Hexanes–EtOAc, 2:1). ^1^H NMR (400 MHz, DMSO-*d*_6_) δ 12.60 (s, 1H), 8.54 (s, 1H), 8.22–8.11 (m, 2H), 7.82 (d, *J* = 8.9 Hz, 1H), 7.24–7.09 (m, 1H). HRMS *m*/*z* calcd. for C_13_H_7_ClF_2_N_2_O [M + H]^+^: 280.0215, found: 280.0215.

#### 4.1.5. 4-Chloro-2-(5,6-dichloro-1*H*-benzo[*d*]imidazol-2-yl)phenol (35889)

This compound was prepared from 4,5-dichlorobenzene-1,2-diamine and 5-chloro-2-hydroxybenzaldehyde using Method B. Yield 92%. TLC R_f_ = 0.30 (Hexanes–EtOAc, 1:1). ^1^H NMR (400 MHz, DMSO-*d*_6_) δ 12.99 (s, 1H), 8.24 (s, 1H), 7.95 (s, 1H), 7.70 (q, *J* = 1.8 Hz, 1H), 7.45–7.40 (m, 2H), 7.08 (dd, *J* = 8.9, 2.4 Hz, 1H). HRMS *m*/*z* calcd. for C_13_H_7_Cl_3_N_2_O [M + H]^+^: 312.9697, found: 312.9696. HPLC: 100% (t_R_ = 3.05 min).

### 4.2. Materials

All experimental compounds were synthesized at Southern Research (Birmingham, AL, USA) and dissolved in DMSO at stock concentrations of 10 mM. Antibodies purchased from Cell Signaling Technologies (Danvers, MA, USA) include phospho-LRP6 (#2568), LRP6 (#2560), cyclin D1 (#2978), survivin (#2808), phospho-4EBP1 (#9451), 4EBP1 (#9452), phospho-STAT3 (Tyr705) (#9145), STAT3 (#4904), Hes1 (#11988), α-tubulin (#2144) and lamin A/C (#2032). Tissue culture media were obtained from ThermoFisher (Waltham, MA, USA) and FBS was obtained from Atlanta Biologicals (Flowery Branch, GA, USA). Niclosamide, insulin, cholera toxin, hydrocortisone, protease and phosphatase inhibitor cocktails were purchased from Sigma-Aldrich (St. Louis, MO, USA). Epidermal growth factor was obtained from PeproTech (Rocky Hill, NJ, USA).

### 4.3. Cell Culture

Nonmalignant mammary cell line MCF10A and TNBC cell lines MDA-MB-231 and MDA-MB-468 were purchased from ATCC (Manassas, VA, USA). MCF10A cells were cultured in DMEM/F-12 (1:1) supplemented with 5% horse serum, 10 µg/mL insulin, 0.5 µg/mL hydrocortisone, 0.02 µg/mL epidermal growth factor, 0.1 µg/mL cholera toxin. MDA-MB-231 and MDA-MB-468 cell lines were cultured in DMEM with 10% FBS. SUM149 and SUM159 cell lines were purchased from Asterand (Detroit, MI, USA) and cultured in DMEM/F12 with 5% FBS, hydrocortisone and insulin. All cell lines were maintained in an incubator at 37 °C and 5% CO_2_.

### 4.4. In Vitro Cytotoxicity

MCF10A and TNBC cells (2 × 10^3^) were seeded with appropriate growth media containing 10% FBS in each well of tissue culture-treated 96-well black plates (Corning #3904) (Corning, NY, USA) 16 h prior to treatment. Cells were treated with SR compounds for 3 days at concentrations of 0–5 µM. Cell viability was then evaluated with Cell Titer Glo (Promega, Madison, WI, USA) and luminescence was measured using a TOPCount NXT plate reader (PerkinElmer, Waltham, MA, USA). Curves were fit according to nonlinear regression and IC_50_ values were calculated using GraphPad Prism 7. Cells were treated in quadruplicates and experiments were performed three times.

### 4.5. Apoptosis Assay

Cells were seeded in 6-well tissue-culture treated plates (Corning) for overnight incubation. Culture media was replaced with growth media containing 1% FBS. Cells were treated with compounds for 48 h prior to harvest. Apoptosis/necrosis was detected by the FITC Annexin V Apoptosis Detection Kit I from Becton Dickinson (San Jose, CA, USA). Cells positive for Annexin V, propidium iodide (PI) or both were all designated as apoptotic.

### 4.6. Western Blot Analysis

Cells were seeded in 6-well tissue-culture treated plates for 48 h until 80% confluency prior to treatment. Whole cell extracts were prepared with Laemmli buffer (5% SDS, 10% glycerol, 0.5 M Tris-Cl) supplemented with protease and phosphatase inhibitors and equivalent amounts of proteins were separated by SDS-PAGE. Proteins were transferred to polyvinylidene difluoride (PVDF) membrane (Bio-Rad, Hercules, CA, USA) and blocked with 5% BSA in tris-buffered saline (TBS) supplemented with 0.1% Tween-20 for 1 h. Membranes were incubated with primary antibody overnight on a shaker at 4 ºC. They were then washed and incubated with secondary antibody for 1 h. Bands were visualized with the Enhanced Chemiluminescence Detection reagents (PerkinElmer).

Cells were lysed using the CERI and CERII reagents of the cytoplasmic-nuclear extraction kit (ThermoFisher), to obtain cytoplasmic fractions following treatment. Nuclear fractions were isolated by using 1× Laemmli buffer and sonication for a few seconds. Membrane was incubated overnight with active β-catenin (MilliporeSigma, Burlington, MA, USA), lamin A/C and tubulin antibodies.

### 4.7. Immunocytochemistry

Cells were seeded on coverslips for 48 h prior to treatment with DMSO and SR compounds for 18 h. Cells were washed with PBS, fixed with 4% paraformaldehyde for 20 min and permeabilized with 0.5% Triton X-100 for 20 min. Following blocking with 3% BSA for 30 min, cells were incubated overnight with active β-catenin antibody (MilliporeSigma) at 4 °C. Cells were incubated with Alexa 488 goat anti-mouse secondary antibody (1:1000) at room temperature for 1 h. To counterstain, cells were incubated with 1:5000 Hoechst 33342 for 20 min. Coverslips were mounted with Prolong Gold Antifade Reagent (ThermoFisher) and cells were observed with Nikon A1R confocal microscope (Nikon Instruments, Melville, NY, USA). Images were obtained at 20× magnification.

### 4.8. Statistical Analysis

Statistical analyses were performed by the unpaired Student *t* test. *p* < 0.05 values were considered significantly different. Results are shown as mean ± SD.

## Figures and Tables

**Table 1:** Scaffold and structures of 2,5-disubstituted phenyl benzimidazoles. Compounds SRI32529, SRI33576, SRI35357, SRI35361 and SRI35889 were evaluated for cytotoxicity and inhibition of Wnt/β-catenin signaling.

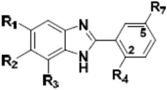					
	R_1_	R_2_	R_3_	R_4_	R_7_
SRI32529	H	NO_2_	H	OH	Cl
SRI33576	H	CF_3_	H	OH	Cl
SRI35357	Cl	H	Cl	OH	F
SRI35361	F	Cl	H	OH	Cl
SRI35889	Cl	Cl	H	OH	Cl

## Abbreviations

BSA	Bovine Serum Albumin
CSC	Cancer Stem Cell
FBS	Fetal Bovine Serum
DMSO	Dimethyl Sulfoxide
ECL	Enhanced Chemiluminescence
ICC	Immunocytochemistry
LRP6	Low-density Lipoprotein Receptor-Related Protein 6
SDS-PAGE	Sodium Dodecyl Sulfate Polyacrylamide Gel Electrophoresis
TCF/LEF	T-cell factor/Lef-1 Family of Transcription Factors
TNBC	Triple-Negative Breast Cancer
